# Recent advances on small molecules in osteogenic differentiation of stem cells and the underlying signaling pathways

**DOI:** 10.1186/s13287-022-03204-4

**Published:** 2022-11-12

**Authors:** Armin Ahmadi, Radman Mazloomnejad, Mohammadreza Kasravi, Babak Gholamine, Soheyl Bahrami, Mohammad Mahdi Sarzaeem, Hassan Niknejad

**Affiliations:** 1 Department of Pharmacology, School of Medicine, Shahid Beheshti University of Medical Sciences, P.O. Box: 1985711151, Tehran, Iran; 2 Ludwig Boltzmann Institute for Experimental and Clinical Traumatology in AUVA Research Center, Vienna, Austria; 3 Department of Orthopedic Surgery, Imam Hossein Medical Center, Shahid Beheshti University of Medical Sciences, Tehran, Iran

**Keywords:** Small molecules, Osteogenesis, Stem cells, Signaling pathway, Bone morphogenic protein, Wnt, Regenerative medicine

## Abstract

Bone-related diseases are major contributors to morbidity and mortality in elderly people and the current treatments result in insufficient healing and several complications. One of the promising areas of research for healing bone fractures and skeletal defects is regenerative medicine using stem cells. Differentiating stem cells using agents that shift cell development towards the preferred lineage requires activation of certain intracellular signaling pathways, many of which are known to induce osteogenesis during embryological stages. Imitating embryological bone formation through activation of these signaling pathways has been the focus of many osteogenic studies. Activation of osteogenic signaling can be done by using small molecules. Several of these agents, e.g., statins, metformin, adenosine, and dexamethasone have other clinical uses but have also shown osteogenic capacities. On the other hand, some other molecules such as T63 and tetrahydroquinolines are not as well recognized in the clinic. Osteogenic small molecules exert their effects through the activation of signaling pathways known to be related to osteogenesis. These pathways include more well-known pathways including BMP/Smad, Wnt, and Hedgehog as well as ancillary pathways including estrogen signaling and neuropeptide signaling. In this paper, we review the recent data on small molecule-mediated osteogenic differentiation, possible adjunctive agents with these molecules, and the signaling pathways through which each small molecule exerts its effects.

**Graphical Abstract:**

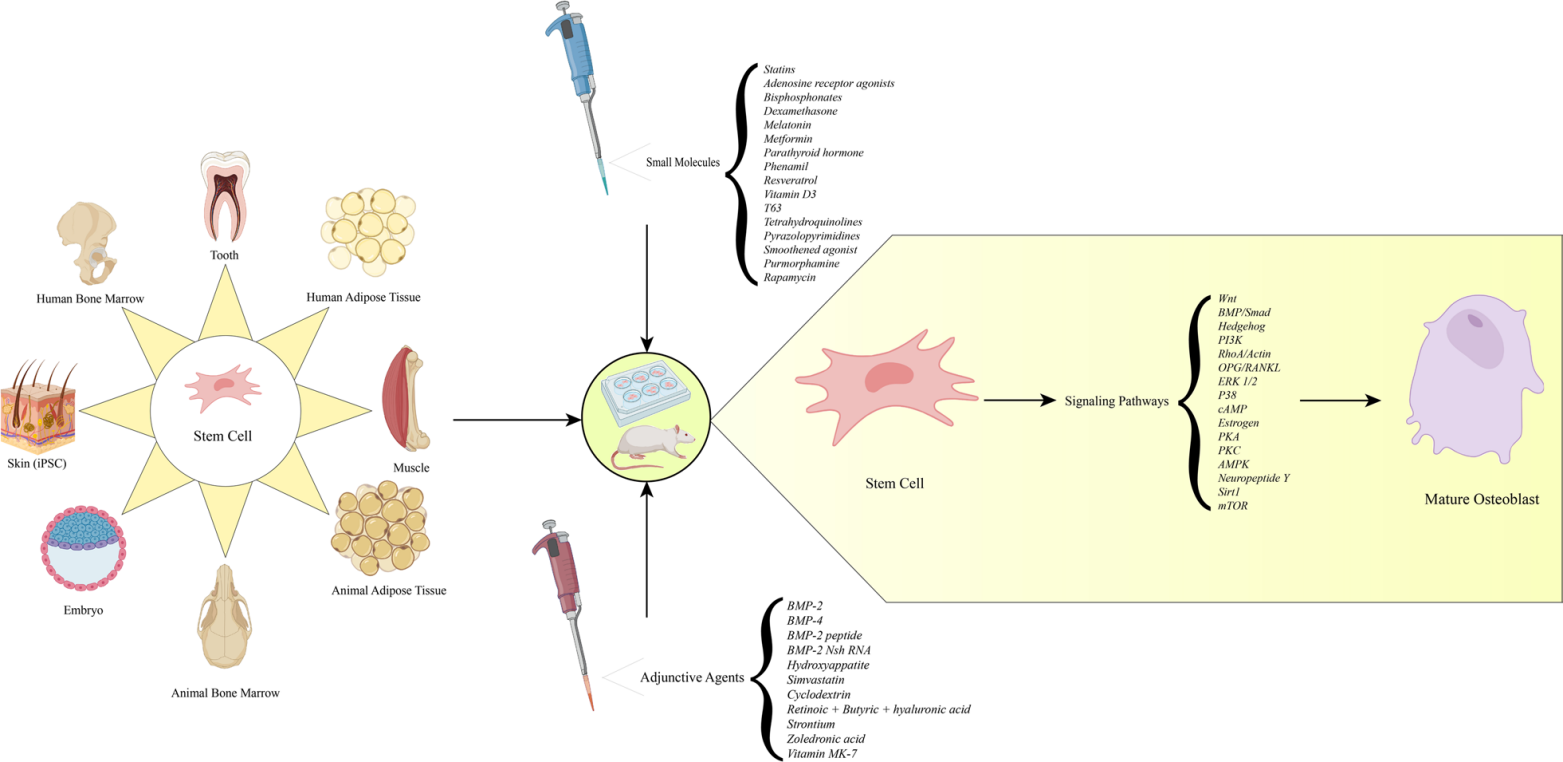

## Introduction

Bone diseases are a principal medical problem for the elderly population which lead to lower quality of life. Osteoporosis is one of these disorders which is becoming increasingly more common, resulting in higher numbers of bone fractures and mortality rates. Bone fractures are more common in women compared to men and cause a variety of complications including decreased mobility and increased long-term care needs. About 1.9%-4.9% of bone fractures in the US have insufficient healing which in turn can be categorized as delayed union or nonunion. Currently, there are no known pharmaceutical agents that single-handedly accelerate nonunion fracture healing. The gold standard of treatment for nonunion fractures is autologous bone grafting. [1–3]. Although autologous bone graft is the gold standard for treatment of many bone defects, the need for a second procedure, risk of infection, and donor site morbidity are some of its disadvantages [4]; therefore, further research to develop safe and effective treatments for healing of these defects is of interest.

Bone tissue engineering proposes a potential means toward acceleration of healing of bone defects [5]. Stem cells are a major factor in tissue engineering and regenerative medicine. These cells are undifferentiated cells that can differentiate into one or more cellular lineages [6]. Self-renewal and vast differentiation capabilities make stem cells a high potential source for tissue engineering [7]. Since there are many similarities between tissue regeneration and development, application of embryological science is considered a handy tool in regenerative medicine [8].

**Fig. 1:**
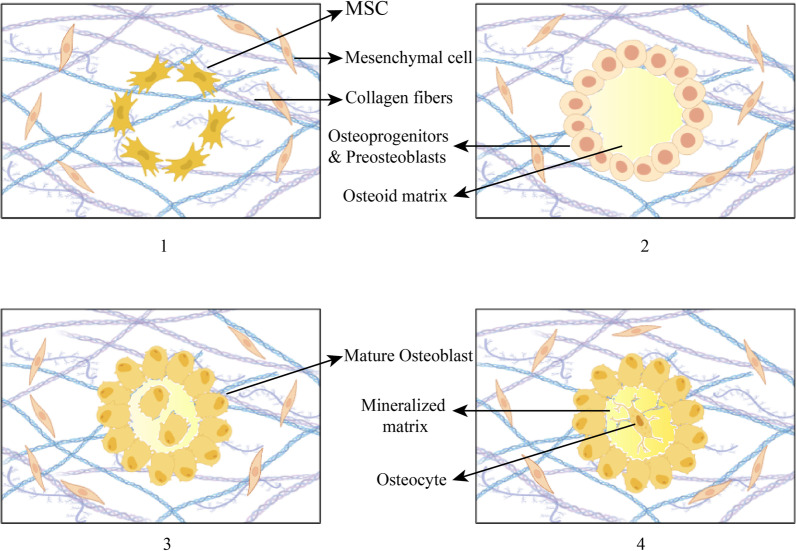
Osteogenic differentiation during intramembranous ossification. 1- MSCs migrate to the site of bone formation and proliferate. 2- MSCs then differentiate into osteoprogenitors and preosteoblasts. 3- These two cell lines deposit the unmineralized bone matrix known as osteoid matrix and differentiate into mature osteoblasts. Osteoid matrix is mineralized by deposition of Ca^2+^. 4- Some osteoblasts are trapped in the mineralized matrix, which later become osteocytes. Expression of the skeletal transcription factors Runx2 and Osterix occurs throughout this process. Also, activation of canonical and non-canonical Wnt signaling as well as BMP/Smad signaling are necessary for various aspects including differentiation, migration and proliferation during intramembranous ossification [11]. Upregulation of the mentioned osteogenic proteins and signaling pathways in the laboratory is often the mechanism through which many small molecules induce osteogenic differentiation of stem cells

In the developmental stages, osteogenesis happens through migration of mesenchymal stem cells to the site in which bone is to be formed. Moreover, the process of bone formation takes place through either of the following methods: direct osteoblast differentiation of cells also known as intramembranous ossification, or indirect bone formation through chondrocyte differentiation followed by later bone formation, also known as endochondral ossification [9]. The majority of osteogenic studies have focused on the former as the main strategy of osteogenic differentiation. In order to achieve osteogenic potential of stem cells, various types of molecules including growth factors have been used (Fig. 1) [10].

The other building block in tissue regeneration is growth factors which are proteins that direct cellular fate into specific paths including differentiation and gene expression. In spite of being major players, growth factors are expensive and usually, high doses are required in order for efficient bone regeneration. For example, bone morphogenic protein 2 (BMP-2) is considered a potent osteoinductive factor (BMPs are among the most widely used growth factors for induction of bone formation). However, treatment with high dose BMP-2 has been reported to have various side effects such as vertebral osteolysis with cyst formation, ectopic bone, cancer and life-threatening inflammatory cervical swelling. One method to overcome the disadvantages of growth factors is utilization of small molecular compounds instead of them. Small molecules have been reported to show great potential in regenerative studies due to low cost, easy membrane passage, and cellular activation. Furthermore, small molecules are generally easier to synthesize and store compared with natural growth factors [12–14]. Another benefit of using small molecules as inducers of differentiation is that they have rapid effects and thus contribute to a more immediate response [15]. In addition, application of small molecules can be used as a method of treatment in order to achieve endogenous stem cell differentiation [16].

Small molecules and growth factors drive stem cells into various lineages through activation of intracellular signaling pathways [17, 18]. Multiple signaling pathways have been found to drive stem cells into osteogenic differentiation [18]. In this paper we review the recent findings on the mechanisms through which small molecules induce osteogenic differentiation, the stem cell types on which these molecules have been found to act, and possible adjuvant agents to enhance osteogenesis of these molecules.

## Intracellular signaling pathways in small molecule-induced osteogenesis

Small molecules exert their osteogenic effects by activating certain signaling cascades in cells. Activation of these cascades, in turn, results in regulations and expression of their specific target genes [18]. Therefore, apprehending how each pathway works is an important step to understand small molecule-mediated osteogenic differentiation.

**Fig. 2:**
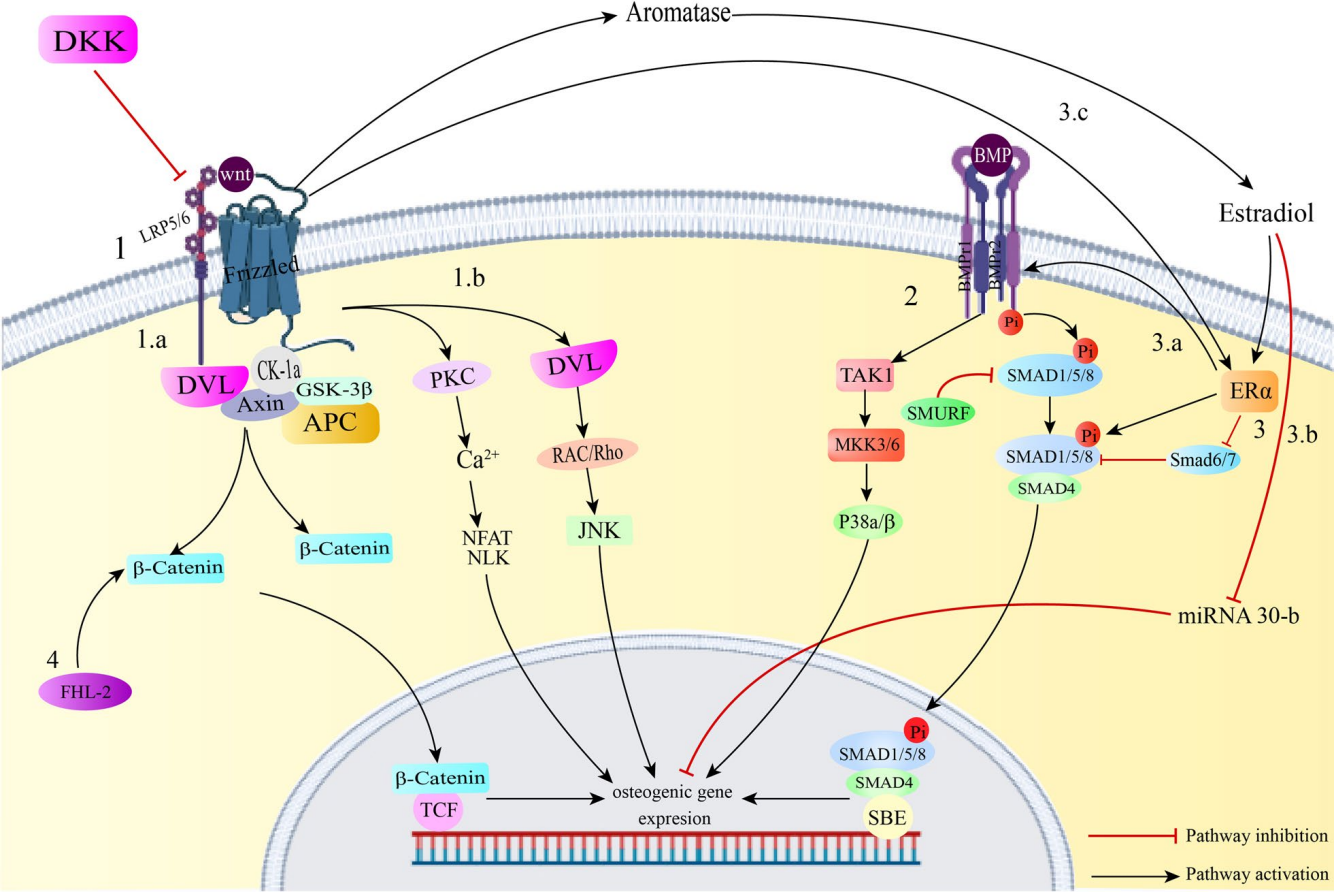
1. The Wnt signaling pathway is initiated by the binding of the specific Wnt ligand to the seven-pass transmembrane Wnt receptors known as Frizzled (FZD). LRP5/6 co-receptors facilitate binding of ligand-receptor which are inhibited by DKK proteins. 1.a. in the canonical Wnt signaling pathway, following binding of the receptor and ligand, Dvl protein is activated which in turn inhibits GSK-3. This results in inhibition of β-catenin protein degradation and rising of nuclear β-catenin concentration which increases osteogenic gene expression. 1.b. the noncanonical Wnt signaling consists of a Ca^2+^ dependent and a Ca^2+^ independent pathway. Activation of both these pathways as well as the canonical Wnt pathway contributes to upregulation of Runx2 gene expression [22]. 2. BMPs are members of the transforming growth factor-beta (TGF-β) superfamily. BMP signaling is initiated as BMPs bind to the heterodimeric Type I/Type II BMP transmembrane receptors. This results in phosphorylation of the receptor Smads (Smad1/5/8) and their binding with Smad4. The Smad complex is then translocated to the nucleus, increasing osteogenic gene expression. Smurf1 is a negative regulator of BMP-Smad signaling which is responsible for degradation and ubiquitination of Smad1 and Smad 5. The Smad-independent BMP pathway also contributes to osteogenesis [23]. 3.a. Estrogen signaling is a signaling pathway known to induce osteogenesis by enhancing BMP signaling. This enhancement is mediated through upregulation of BMP type II receptor and Smad1/5/8 as well as inhibition of the inhibitory Smad6/7. 3.b. Moreover, estradiol has been found to exert an inhibitory effect on miRNA 30-b. This miRNA is an inhibitor of the Runx2 gene [24]. 3.c. Estrogen signaling is also known to induce osteogenesis through crosstalk with Wnt signaling pathway. The mechanism suggested for this effect is thought to be upregulation of two key elements in estrogen signaling. Wnt ligands upregulate the estrogen receptors as well as aromatase, the important enzyme in estrogen synthesis. 4. Four and a half LIM domains protein 2 (FHL-2), is a transcriptional coregulator crucial for osteogenic differentiation, which is increased by dexamethasone. Increasing FHL-2 levels contributes to nuclear translocation of β-catenin protein and enhancement of subsequent gene transcription [25]

Many signaling pathways are known to induce osteogenesis in stem cells [18]. One of these pathways is Wnt signaling, which is a well-established pathway in osteogenic differentiation. This pathway consists of two distinct intracellular cascades known as canonical (β-catenin-mediated) and noncanonical Wnt paths, both of which are known to induce osteogenic differentiation (Fig. 2). There are a total of 19 Wnt ligands found in humans which bind to the specific seven-pass transmembrane Wnt receptors known as Frizzled (FZD). The end-product of the canonical Wnt signaling is nuclear translocation of β-catenin protein and manipulation of its target genes. When Wnt ligands are absent, β-catenin is degraded by an intracellular complex consisting of glycogen synthase kinase 3 (GSK-3). Activation of canonical Wnt results in activation of Disheveled (Dvl) protein which then inhibits GSK-3. Although Noncanonical Wnt signaling is not as well characterized as the β-catenin-mediated Wnt pathway, it can also play a role in bone tissue differentiation. Noncanonical Ca^2+^ dependent Wnt pathway contributes to osteogenesis of stem cells. This pathway is triggered by binding of Wnt ligands to the FZD receptors. Then, activation of G protein results in Ca^2+^ ions being released from the endoplasmic reticulum, which results in initiation of protein kinase C (PKC) signaling pathway. Wnt-5a is a noncanonical Wnt ligand which has an important action in osteogenesis of BMP-2. It is known that Ca^2+^ dependent Wnt signaling is involved in osteogenesis of Wnt-5a [19–21]. There are a group of co-receptors in Wnt signaling known as low-density lipoprotein receptor-related proteins 5 or 6 (LRP5/6) which facilitate the binding of Wnt ligands and FZDs. Binding of LRP5/6 inhibitors (known as DKK) to the LRP5/6 co-receptors results in disruption of Wnt ligand-FZD binding, and thus inhibiting Wnt signaling [19].

BMP/Smad signaling is considered another important osteogenic signaling pathway. BMP/Smad signaling is known as a pro-osteogenic, pro-adipogenic signaling pathway [18]. This pathway is initiated by binding of the BMPs, a group of proteins of the TGF-β superfamily, to the heterodimeric Type I/Type II BMP transmembrane serine/threonine kinase receptors (Fig. 2) [26]. Then, either a Smad-dependent or a Smad-independent cascade is started intracellularly. In the Smad-dependent pathway, binding of the BMP ligand to its specific receptor results in phosphorylation and binding of the Receptor-Smads (Smad1/5/8) to the Common-Smad (Smad4). Translocation of this complex to the nucleus, in turn results in modulation of BMP target genes and osteogenic differentiation [27].

**Fig. 3:**
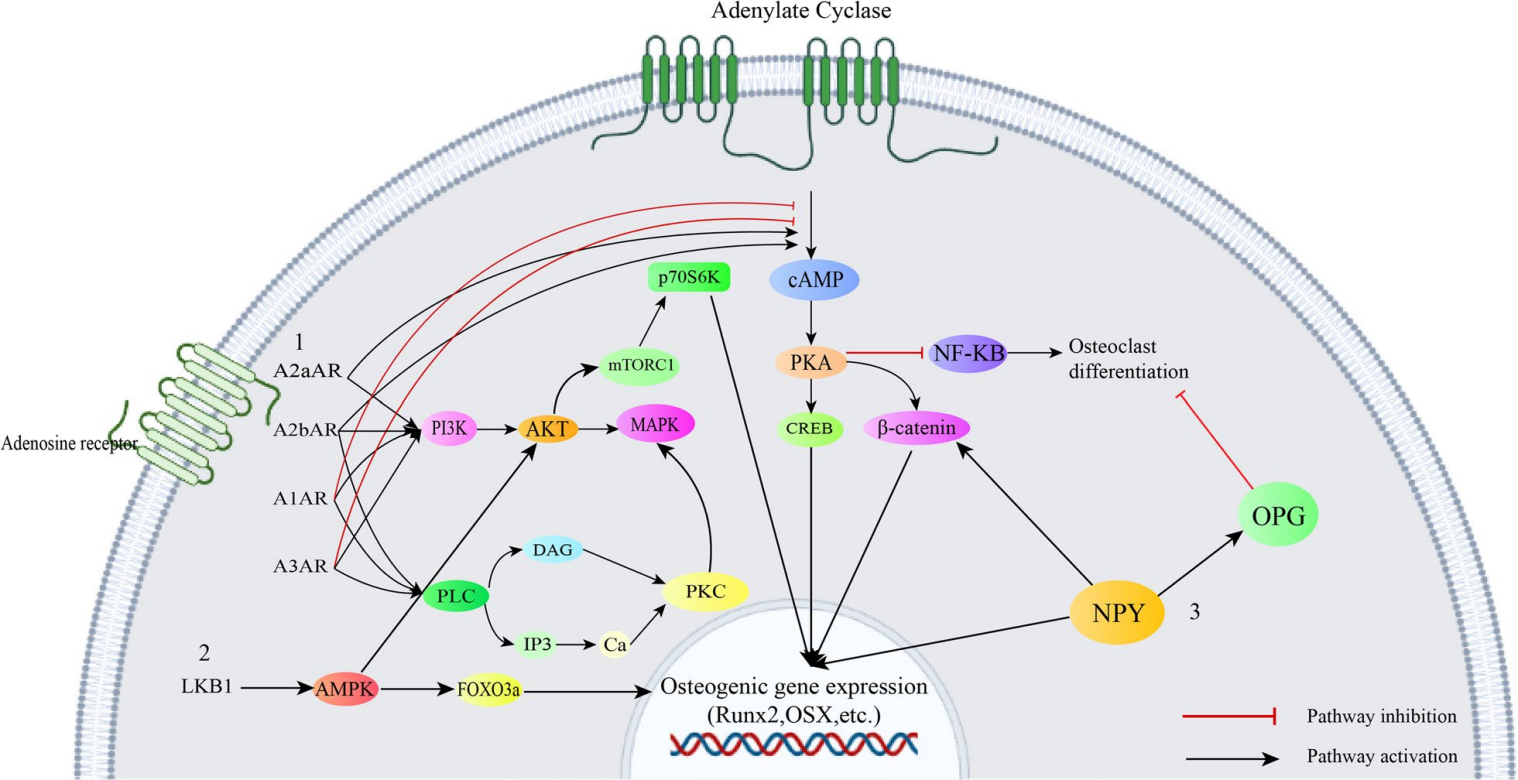
1. A2a and A2b adenosine receptors activate the adenylate cyclase enzyme by Gαs subunit. A1 and A3 receptors inhibit this enzyme by Gαs subunit. All four of the mentioned receptors activate PI3K signaling. All four activate PLC signaling except for A2a receptor [32]. Activation of the A1 adenosine receptor results in upregulation of Dvl and p-GSK-3β, which finally leads to increased canonical wnt signaling and osteogenesis. Activation of adenylate cyclase by adenosine receptors results in engagement of the cAMP-PKA pathway and Runx2 gene expression [33]. This pathway also contributes to suppression of NF-KB, which is an osteoclastogenic factor [34]. 2. LKB1, as an AMPK kinase, activates AMPK. Activation of AMPK results in Runx2 gene expression through AMPK/FOXO3a/Runx2 pathway. Also, AMPK activation can stimulate Akt-mTORC1-p70S6K pathway [35]. This is known as the pathway through which mTORC1 signaling exhibits osteogenic differentiation [36]. 3. NPY signaling influences osteogenesis by 3 pathways. First, NPY signaling upregulates Runx2 and thus directly mediates osteogenesis. Second, this signaling initiates crosstalks with Wnt-β-catenin signaling pathway. NPY signaling results in upregulation of β-catenin and GSK-3β levels and enhances canonical Wnt signaling. Finally, by rising OPG levels, NPY signaling blocks osteoclastogenesis by negatively regulating OPG/RANKL/RANK pathway

Unlike the BMP signaling pathway, Adenosine-signaling is a pro-osteogenic, anti-adipogenic signaling pathway [28]. The relationship between extracellular ATP and adenosine and their receptors is responsible for many healthy or pathological procedures. Adenine-based purines have two types of receptors; P1 receptors which mainly respond to adenosine and P2 receptors that are activated by purine as well as pyrimidine bases. A1, A2a, A2b and A3 are four subtypes of P1 adenosine receptors (Fig. 3). It was previously known that extracellular adenosine could enhance osteogenic differentiation through several P2Y and P2X receptors. Recently, several of the P1 receptors have also demonstrated roles in bone formation. The A2B adenosine receptor is a Gs/q-protein-coupled receptor which is activated when the cellular levels of adenosine are within micromolar ranges. This receptor is also expressed in mesenchymal stem cells (MSCs) and osteoblast progenitors. Bone injury results in increased extracellular ATP and oxidative stress and inflammation contribute to increased A2B adenosine receptor expression [29–31].

**Fig. 4:**
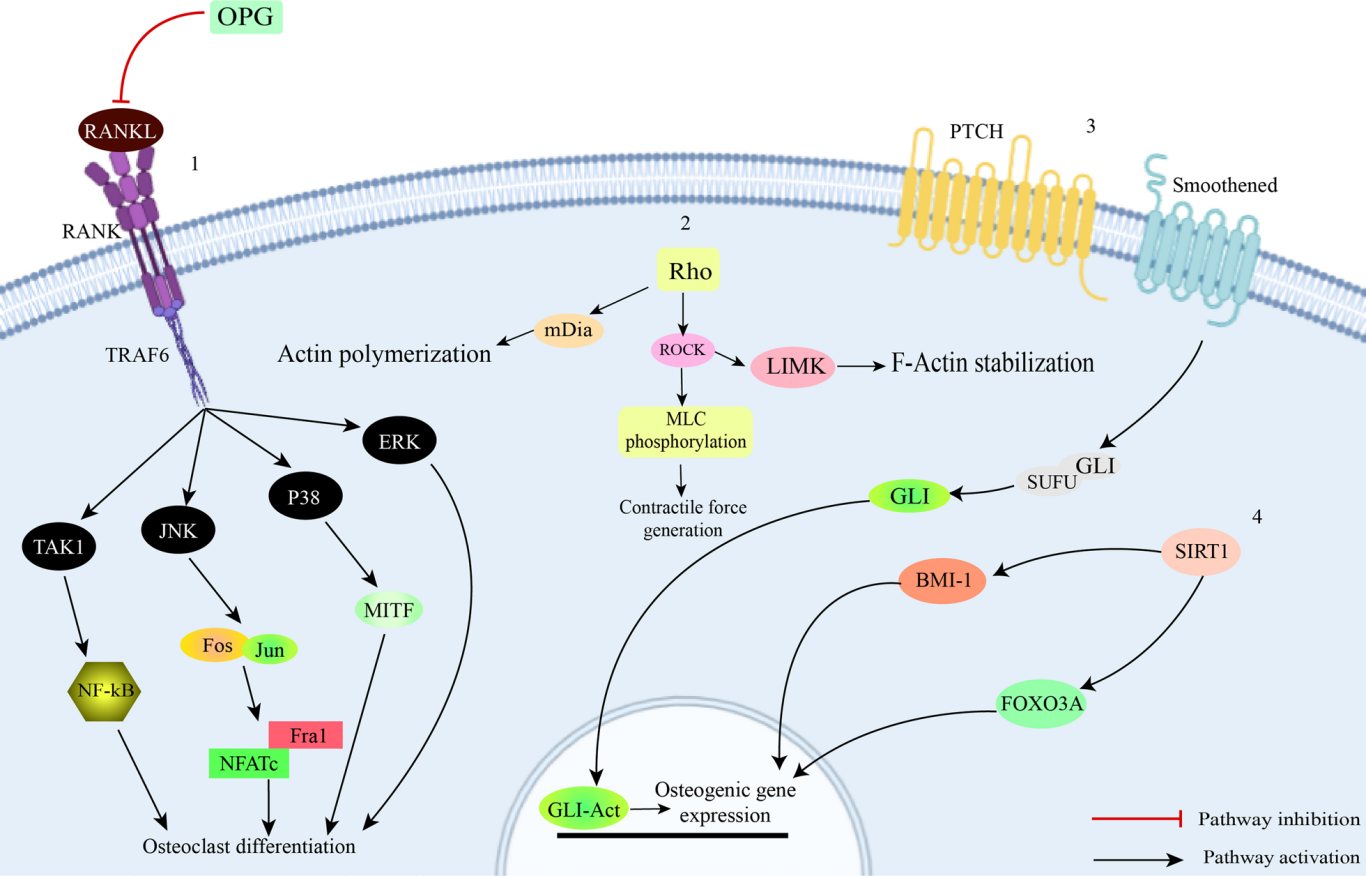
1. Osteoprotegerin (OPG) inhibits binding of RANKL to RANK. RANKL-RANK binding results in trimerization of the receptor. This, in turn triggers activation of tumor necrosis factor receptor-associated factor (TRAF)-6 and activation of the subsequent pathways. All of the above-mentioned steps are necessary for osteoclast differentiation. High concentrations of OPG result in inhibition of the pathway and thus negatively regulate osteoclast differentiation [39]. 2. Rho is a family of proteins which act in actin polymerization. Activation of this pathway results in activation of Rho-associated coiled coil-containing protein kinase (ROCK) and mammalian Diaphanous (mDia). Furthermore, ROCK is known as the phosphorylator of LIM motif-containing protein kinase (LIMK). LIMK is strongly associated with bone mass and osteoblast differentiation. Therefore, initiation of Rho signaling pathway positively regulates osteogenic differentiation. 3. Molecules that activate the hedgehog signaling pathway bind to patched receptor (PTCH). Without the presence of hedgehog ligands, The PTCH inhibits Smoothened (SMO). Ligand-PTCH binding results in Termination of this inhibition. Followed by this, glioma-associated oncogene transcription factor (GLI) is released and translocated into the nucleus which increases transcription of target genes [40]. 4. Sirt1 is a deacetylator of various transcription factors. FOXO3A is one of the transcription factors it acetylates. FOXO3A is a key regulator of stress resistance gene transcription which also mediates osteogenic differentiation. Another downstream pathway of Sirt1 is Bmi-1. Sirt1 activation has been found to result in increased binding of Sirt1 and Bmi-1 and thus contributing to osteogenic gene expression

Similar to adenosine signaling, Hedgehog (Hh) signaling is known as a pro-osteogenic, anti-adipogenic signaling pathway [37]. This signaling was first found in the fruit fly *Drosophila* and consists of three members: Desert Hedgehog, Indian hedgehog and Sonic hedgehog. Hedgehog signaling is initiated by binding of the hedgehog ligand to the transmembrane protein receptor known as Patch (PTCH) (Fig. 4). This binding results in inhibition of the seven-pass transmembrane protein Smoothened (Smo) suppression and subsequently the Gli transcription factor family is activated. Activation of the Gli transcription factors in turn leads to expression of Hedgehog target genes and osteogenic differentiation [38].

Protein kinases are a group of enzymes that are responsible for phosphorylation of proteins. They are generally known as on/off switches of various cellular functions including differentiation [41]. Mitogen-activated protein kinases (MAPKs) are a family of well-established protein kinases which are generally known as important players in cell differentiation and proliferation. Several studies have been conducted regarding the effect of p38 and Extracellular receptor kinase 1/2 (ERK 1/2) on osteogenic differentiation and contradictory results have been found to date. This suggests that the effect of either of two protein kinases might depend on several cues such as cell type, stage of development and culture system. Regardless, it is known that these two protein kinases are involved in osteogenic differentiation of stem cells [42].

The PKC family includes 15 molecules which are divided into 3 different groups based on the substrate required for their activation. Certain members of this family are known to exert different effects on hard tissue regeneration; e.g., PKC δ and β are considered pro-osteogenic kinases, whereas PKC α and μ downregulate osteogenesis [43]. Protein kinase A (PKA) is known to regulate various cellular functions including the immune response and lipid metabolism. It is also known as a downstream substrate of the BMP signaling pathway. Downregulation of a PKA inhibitor is found to be necessary for BMP-2 mediated osteogenesis. PKA is responsible for regulation of RUNX2, osteocalcin and osterix which are important proteins in the process of bone regeneration (Fig. 3) [44].

Adenosine monophosphate-activated kinase (AMPK) signaling is known to be responsible for maintaining cellular energy homeostasis. This signaling pathway has been found to be an important factor in bone formation and inhibition of adipogenic differentiation. Chava et al. have recently demonstrated that activation of AMPK results in phosphorylation of RUNX2 at serine 118. The phosphorylation of RUNX2 is crucial for osteogenic differentiation and dephosphorylation of AMPK results in adipogenic differentiation. These results indicate that AMPK phosphorylation modulates homeostasis between osteogenic and adipogenic differentiation and shifts stem cells towards osteogenic lineages [45] (Fig. 3).

Several other signaling pathways have been found to be responsible in small molecule mediated osteogenesis. These pathways include Estrogen receptor (ER) signaling, RhoA/actin, RANKL/RANK/OPG, mTOR signaling and neuropeptide signaling [46–50].

One of the pathways responsible for fracture healing is neuropeptide Y (NPY) signaling (Fig. 3). Previously known as a food intake regulator, NPY is now also known as an important bone remodeling regulator. NPY signaling induces osteogenic differentiation through increasing Runx2 level [51]. Also, certain crosstalks between NPY signaling and Wnt signaling contribute to osteogenesis through NPY signaling. Engagement of NPY signaling results in an increase in β-catenin and GSK-3β [52]. Apart from affecting bone formation, NPY is also known to influence bone resorption. It has been found that NPY upregulates Osteoprotegerin (OPG) production in bone marrow mesenchymal stem cells (BMMSCs) and thus inhibits osteoclastogenesis through OPG/RANKL/RANK pathway [46, 51].

Osteoprotegerin (OPG), Receptor activator of NF-kB ligand (RANKL) and Receptor activator of NF-kB (RANK) are the main components of the osteoclast maturation RANKL/RANK/OPG signaling pathway. Osteoclast differentiation and bone resorption are mainly modulated by secretion of OPG and RANKL. Binding of OPG to RANK can block binding of RANKL to RANK; this means that an increase in OPG/RANKL ratio can block osteoclast differentiation and bone resorption [49]. It is also known that increasing OPG/RANKL ratio can lead to osteogenic differentiation of stem cells [53] (Fig. 4).

RhoA/actin signaling is another pathway found to be responsible in osteogenesis of stem cells. Rho GTPase family is the most important modulator of actin cytoskeleton rearrangement. RhoA is one of the twenty Rho proteins which plays a role in myosin 2 activation and production of actomyosin force and stress fiber. It is known that formation of stress fibers and stabilization and polymerization of actin shifts stem cell fate toward osteogenic ends [48] (Fig. 4).

Mechanistic target of rapamycin (mTOR) signaling is a nutrient-sensing pathway which plays roles in cell cycle progression and survival. This pathway consists of two separate complexes known as mTORC1 and mTORC2, each of which is responsible for certain cellular functions. It has been recently found that mTOR signaling plays an important role in osteogenic differentiation of stem cells (Fig. 3). However, contradictory results have been found regarding whether activation or inhibition of this pathway promotes bone tissue regeneration. It has been suggested that these conflicting results might be due to stage-specificity of mTOR activation in osteogenesis [50, 54].

Another pathway which has recently been found to play an important role in osteogenesis is estrogen signaling (Fig. 2). Estrogen receptor-alpha (ER-alpha) and G protein coupled estrogen receptor-1 (GPER-1) are two types of estrogen receptors found to be effective in bone regeneration [55]. One pathway with a notable crosstalk with Estrogen receptor signaling is the Wnt pathway. It has been found that activation of ER signaling results in enhancement of osteogenic differentiation mediated by Wnt-3a. This effect is thought to be due to Wnt-3a-induced upregulation of ER-alpha and aromatase. Aromatase is a key enzyme in estrogen synthesis [47]. Estrogen signaling is also known to exhibit crosstalk with BMP signaling. Estradiol increases Smad1/5/8 and BMP type II receptors. Estradiol also inhibits the inhibitory Smad6/7 [56]. As mentioned above, crosstalks between osteogenic signaling pathways account for osteogenic differentiation. The above-mentioned cascades are the most extensively studied cascades on which small molecules influence, leading to osteogenesis. In the next section, we assess each small molecule individually with an eye for the pathways through which these agents induce osteogenesis.

## Small molecules

### Statins

Statins are a group of cholesterol lowering drugs which primarily target HMG-CoA reductase. They are also known as osteo-inductive agents [57]. Simvastatin (SM) is a highly lipophilic statin which causes this drug to pass through the cell membrane in a more facilitated fashion compared with its other counterparts including pitavastatin and mevastatin [58]. Thus, it is the most widely used statin for the purposes of bone regeneration. Pagkalos et al. studied the effects of SM on osteogenic differentiation of murine embryonic stem cells (ESCs). They added SM to a traditional osteogenic medium containing ascorbic acid, β-glycerophosphate, and dexamethasone which resulted in mineralized matrix formation. However, no dose-dependent effect of SM was observed in this matter. Moreover, to evaluate osteoinductivity of SM, the cells were cultured with SM without presence of the aforementioned osteogenic medium. It was found that SM could alone induce osteogenesis of ESCs and that the optimal dose for this was 0.1 nM. Higher dosages of the drug in the micromolar range were found to significantly lower viability of the cells [58]. ESCs are not the only type of stem cells that can be differentiated by Statins. Niu et al. studied the effects of SM on osteogenic differentiation of human bone marrow mesenchymal stem cells (hBMMSCs) and its possible immune-modulatory outcomes. They found that SM shifted BMMSCs towards osteogenic lineages. To assess whether SM affects immunogenicity, BMMSCs were first seeded with SM for one week and followed by incubation with allogeneic peripheral blood mononuclear cells (PBMCs). It was found that SM had no apparent effect on proliferation of PBMCs. Therefore, no significant effect was observed in regards to SM changing BMMSC immunogenicity. Furthermore, when phytohemagglutinin was added as a PBMC proliferation stimulator, addition of SM did not seem to oppose inhibition of PBMC proliferation caused by BMMSCs. Thus, culturing of BMMSCs with SM did not affect immunosuppressive effects of these types of stem cells [59].

Several signaling pathways are thought to be related with statin-mediated osteogenesis. Zhang et al. found that addition of Dickkopf-1(DKK-1), a Wnt inhibitor, to the medium one hour prior to SM, can significantly downregulate osteogenic differentiation of rat BMMSCs. This drug was also found to increase expression of β-catenin protein. Furthermore, shRNA mediated inhibition of β-catenin protein expression attenuated SM-induced osteogenesis. These findings suggest that statins activate Wnt/β-catenin signaling pathway to achieve osteogenic differentiation [60]. There is clear evidence that statins can activate β-catenin-independent Wnt signaling as well. It was found that Frizzled-2 and Wnt-5a, which are both known as members of noncanonical Wnt signaling, were upregulated during treatment of MSCs with SM [61].

Estrogen signaling is another pathway which plays role in stem cell differentiation by statins. Chuang et al. studied the interconnection of statin-related osteogenic differentiation and estrogen signaling in mouse bone marrow MSCs. They utilized fulvestrant as an estrogen receptor antagonist to block estrogen signaling. It was found that blockade of ER signaling downregulated SM-mediated osteogenesis; however, mRNA expression of ER-alpha was not altered. This suggests that SM administration does not affect gene expression of ER-alpha, but increased ER function. To further elucidate whether ER-alpha and/or GPER-1 is responsible for SM mediated osteogenesis, ER-alpha and GPER-1 were inhibited by their specific antagonists. While it was found that GPER-1 antagonism did not affect osteogenesis, ER-alpha antagonism significantly reduced osteogenic markers and BMP-2 expression induced by SM. Furthermore, it was found that SM can act as a weak receptor agonist for ER-alpha [55].

BMP/Smad signaling is found to be another pathway responsible for statin mediated osteogenic differentiation. SM can increase Smad1/5/8 protein phosphorylation level and reverse TNF-α related inhibition of Smad1/5/8 phosphorylation in MC3T3-E1 cells [62]. Also, application of SM has been found to result in an increase in BMP-2 levels in stem cells undergoing osteogenic differentiation [63]. While statins can engage the pro-osteogenic/pro-adipogenic BMP pathway [18], they can also activate the pro-osteogenic/antiadipogenic Rho/actin/cell rigidity pathway to induce osteogenesis. Tai et al. found that SM increases levels of active RhoA in MSCs during osteogenesis. It was further noted that interfering with actin cytoskeletal organization and loss of cell rigidity downregulate osteogenic differentiation of SM both in vivo and in vitro. Therefore, RhoA/actin/cell rigidity pathway might also be an important pathway in osteogenic differentiation of statins [64]. Niu et al. investigated whether ERK1/2 pathway is involved in osteogenesis mediated by SM. To do so, they used SM to differentiate human osteoblast-like MG63 cells and measured ERK1/2 phosphorylation levels at different time points throughout the experiment. They report that although SM administration resulted in activation of ERK1/2 in the first 24 h, from 24 h until 7 days, inhibitory effects were observed. To test whether ERK1/2 plays a role in osteogenic differentiation of SM, they added an ERK1/2 inhibitor. Addition of the specific ERK1/2 inhibitor resulted in higher levels of osteogenic-specific proteins in the cultures [65]. The effects of p38 MAPK have also been studied in SM-mediated osteogenesis. p38 MAPK is found to be upregulated in cultures of rat BMMSCs which have been incubated with SM. Also, blocking of p38 results in lower levels of osteogenic gene expression and matrix mineralization [66]. Since it results in better nutrient and oxygen supplementation, and provides a route for stem cells to reach the zone of bone defect, enhanced angiogenesis has been reported to have a close connection with osteogenesis. Statins can mediate angiogenesis by increasing gene expression of vascular endothelial growth factor (VEGF) and fibroblast growth factor (FGF-2). Therefore, statins can contribute to osteogenesis not only through osteogenic-specific pathways but also through angiogenesis [67].

Several studies have focused on combination of treatment with statins and other osteogenic agents or materials and whether this combination can enhance osteogenic differentiation [55, 57, 62, 63, 68]. Co-administration of SM and hydroxyapatite is one of the investigated methods. Hydroxyapatite synergistically enhances osteogenic differentiation of SM in dental pulp stem cells (DPSCs). This synergistic effect is more significant in lower concentrations of hydroxyapatite. These results suggest that employment of hydroxyapatite is a possible means of adjuvant treatment with SM [57]. Tao et al. investigated combined treatment with SM and parathyroid hormone (PTH). It was found that co-treatment with SM and PTH causes bone healing around hydroxyapatite-coated titanium implants after osteoporotic fractures. This healing was significantly enhanced when both agents were utilized compared to single treatment of each one. Therefore, PTH is considered a potential additive agent in SM induced osteogenic differentiation [68]. BMP-2 is another osteogenic molecule considered as a possible adjuvant agent when utilizing SM. The effects of SM- BMP-2 combined treatment on osteogenic differentiation of murine osteo-precursor cells was investigated in another study. It was found that concurrent usage of BMP-2 and SM synergistically induced osteogenesis through enhancement of phosphorylated Smad1/5/8. Therefore, addition of BMP-2 to SM is considered a possible strategy to increase bone formation [62]. SM is known to enhance osteogenic differentiation by BMP/Smad pathway. However, the osteoinductive effects of this pathway can be reversed by the specific BMP antagonist, Noggin. Thus, suppression of Noggin expression may enhance SM mediated osteogenic differentiation. Huang et al. studied the effects of co-delivery of SM and si-RNA targeting Noggin gene (Nsi-RNA). Combination of the two agents resulted in higher expression of BMP-2 and lower expression of Noggin. The BMP-2/Noggin ratio increased with the Nsi-RNA concentration in a dose-dependent manner. As a result, application of SM and Nsi-RNA significantly increased osteogenic effects both in vitro and in vivo. Thus, application of Nsi-RNA alongside SM is considered a possible adjuvant treatment for osteogenic differentiation [63, 69]. Chuang et al. investigated the effects of SM and estradiol co-treatment on osteogenic differentiation of D1 cells. Combination of SM and estradiol was found to increase tissue mineralization compared with either of the components alone. These results indicate that co-administration of SM and estradiol can be considered a potential method in order to improve bone tissue engineering [55]. Graphene oxide is another agent which has recently been found to enhance osteogenic effects of SM. Oh et al. demonstrated that a complex made up of graphene oxide and SM shifted mouse BMMSCs into osteogenic lineages. Furthermore, the complex exhibited higher levels of osteogenic proteins and tissue mineralization compared to SM alone [70].

### Adenosine receptor agonists

The small molecule adenosine is a purine nucleoside, widely used as a vasodilator and neuromodulator. Recently, a growing body of attention has been focused on adenosine and adenosine receptors as factors implicating in bone regeneration [28]. Exogenous adenosine supplementation is an effective method in order for successful osteogenic differentiation of mouse BMMSCs [71]. Also, administration of exogenous adenosine as a differentiation factor to cultured human ESCs has enhanced osteogenesis through A2b receptor adenosine signaling. In a study performed by kang et al. investigating osteogenic differentiation of pluripotent stem cells (PSCs) induced by single treatment of adenosine, it was found that unlike other adenosine receptors, A2b receptor was upregulated in treatment group. Exogenous adenosine is not the only adenosine receptor agonist which has been studied in regards to osteogenesis. Several studies in this field have been done focusing on using specific adenosine receptor agonists. Stimulation of A2b receptor by its specific agonist has been reported to induce osteogenic differentiation and in vivo bone formation [28, 29]. In another study, A2b receptors were studied more thoroughly. Barresi et al. investigated combinations of an orthosteric A2b receptor activator with several allosteric A2b receptor activators on human BMMSCs osteogenic differentiation. Results of the assay revealed that combination of the two types of agonists improved tissue mineralization [72].

Although not quite as much as A2b receptor, A1 adenosine receptors have also been found to contribute to increase in bone tissue formation. Activation of A1 receptor has been found to be connected with activation of MAPK and PI3K pathways [30]. Moreover, it seems that there is a crosstalk between A1 adenosine receptor signaling and Wnt signaling regarding osteogenic differentiation of DPSCs. Utilization of an A1 adenosine receptor agonist led to upregulation of Dvl and GSK-3β, both of which are members of the canonical Wnt signaling pathway (Fig. 2). Also, employment of DKK-1 as a Wnt antagonist and si-RNA mediated suppression of Dvl inhibited osteogenic differentiation of DPSCs under similar conditions. Thus, A1 adenosine receptor signaling promotes osteogenesis at least in part through Wnt pathway [30]. In addition to A1, A2b adenosine receptor signaling is capable of osteoinduction. MSCs obtained from A2b adenosine receptor knockout mice reveal a reduced osteogenic marker expression compared with wildtypes when cultured in osteogenic media. Additionally, induction of A2b adenosine receptor signaling by the receptor’s specific ligand in wildtype cells is known to increase osteogenic markers. A2b adenosine receptor signals through cAMP; a pathway which is known to mediate MSC differentiation into skeletal lineages. It was observed in this study that cAMP levels were increased in wildtype cells cultured with A2b receptor agonist. Also, a cAMP analog was used in order to simulate bypassing of the receptor. It was found that even when the ligand is absent, in the presence of a cAMP analog, osteogenic gene expression is positively regulated (Table 1). This confirms that cAMP signaling is at least partially responsible for A2 adenosine receptor ligand mediated osteogenesis (Fig. 3) [29].

**Table 1:** Summary of osteogenic small molecules, signaling pathways, adjunctive agents and cell lines on which they were evaluated

Molecule name	Mechanisms (Signaling cascades)	Adjuvant treatments	Cell lines
Statins	⊕ Canonical & non-canonical Wnt ⊕ Estrogen Signaling ⊕ BMP/Smad ⊕ RhoA/actin ⊖ ERK ½ ⊕ P38	Hydroxyapatite PTH BMP-2 Nsi-RNA Estradiol Graphene Oxide	Murine ESC hBMMSC Rat BMMSC MC3T3-E1 MG-63 DPSC
Adenosine	⊕ PI3K pathway ⊕ Canonical Wnt ⊕ cAMP signaling	-	Mouse BMMSC hESC hiPSC hBMMSC DPSC
Bisphosphonates	⊖ OPG/Rankl ⊖ mTOR ⊕ BMP/Smad	Simvastatin BMP-2	Mouse BMMSC MG-63 Rat AD-MSC MC3T3-E1 Rat BMMSC
Dexamethasone	⊕ Canonical Wnt ⊕ ERK ½	BMP-2 BMP-2 peptide Hydroxyapatite	SHED Rat BMMSC Rat muscle-derived stromal cell Canine iPSC-derived MSC AD-MSC
Melatonin	⊕ AMPK ⊕ PKA signaling ⊕ PKC signaling ⊕ Neuropeptide Y signaling ⊕ Canonical Wnt ⊕ BMP/Smad signaling ⊕ P38 ⊕ ERK ½ ⊖ OPG/Rankl	Cyclodextrin Retinoic + Butyric + hyaluronic acid BMP-4	Rat BMMSC hBMMSC C2C12 Rabbit DPSC MC3T3-E1 DPSC
Metformin	⊕ AMPK ⊕ Canonical Wnt ⊕ BMP/Smad ⊖ ERK ½ ⊖ P38	-	hCV-MSC MC3T3E1 hiPSC-derived MSC hBMMSC periodontal ligament SC UC-MSC
Phenamil	⊕ BMP/Smad signaling	BMP-2 Nsh-RNA Strontium	AD-MSC MC3T3-E1 DPSC hBMMSC
Parathyroid Hormone	⊕ BMP/Smad signaling ⊕ cAMP ⊕ PKC signaling ⊕ PKA signaling	Vitamin K	Rat BMMSC hBMMSC hAD-MSC Mouse BMMSC
Resveratrol	⊕ ERK ½ OPG/Rankl ⊕ Sirt1 ⊕ Canonical Wnt	Zoledronic acid	hBMMSC MC3E3-T1 DPSC hESC-derived MSC progenitor Canine AD-MSC Mouse BMMSC Canine BMMSC
Vitamin D3	⊕ Canonical Wnt ⊕ Sirt1	BMP-2 Vitamin MK-7	hiPSC (105) MC3T3-E1 hBMMSC mouse BMMSC hAD-MSC
T63	⊕ Canonical Wnt ⊕ BMP/Smad	-	MC3T3-E1
Tetrahydroquinoline	⊕ Canonical Wnt	-	C2C12
Pyrazolopyrimidine	⊕ Canonical Wnt	-	C2C12
Smoothened agonist	⊕ Hedgehog	-	Mouse ESC Mouse iPSC hiPSC
Purmorphamine	⊕ Hedgehog	-	Rat BMMSC DPSC
Rapamycin	⊖ mTOR		BDSC

⊕ :activation of the pathway by small molecule ⊖ : inhibition of the pathway by small molecule

### Bisphosphonates

Bisphosphonates (BPs) are a drug class commonly used as treatment of osteoporosis, Paget’s disease and malignancy-related hypercalcemia due to being able to decrease the process of bone resorption. Although BP administration has resulted in improvement of osteointegration and implant stability, its application has been limited due to osteonecrosis of jaw. One of the safer techniques which is used in order to limit such side effects is pretreatment of cells with BPs. Wen et al. studied pretreatment of BMMSCs with zoledronic acid (ZA) in order to develop a method through which this complication could be avoided. Pretreatment of stem cells with BPs before implantation has been suggested as a possible means through which osteonecrosis can be avoided. To evaluate the outcomes of ZA pretreatment, BMMSCs were pretreated with ZA and then cultured in an osteogenic medium. It was observed that 0.5 and 1 Micromolar of ZA lead to enhancement of osteogenic differentiation. Furthermore, it was found that unlike high dose, low dose ZA administration resulted in an increase in proliferation of BMMSCs in the first 72 h of treatment. Moreover, they investigated the mechanism through which ZA leads cells towards osteogenesis. They found that osteogenic induction of stem cells with ZA significantly increased OPG without altering Rankl level. This leads to an increase in OPG/Rankl ratio [73]. In another study by Gao et al., it was found that ZA regulates osteoblastic differentiation through mTORC1 pathway. This signaling pathway is an important part during osteogenic differentiation. To study the pathway through which ZA induces osteogenesis, they seeded MG63 pre-osteoblast cells with different doses of ZA. It was observed that lower doses of ZA resulted in lower mTORC1 activity which led to higher expression of osteogenic proteins. On the other hand, higher doses of ZA contributed to higher mTORC1 activity and lower levels of osteogenic markers. Moreover, co-incubation of cells with ZA and the mTORC1 inhibitor rapamycin significantly downregulated both mTORC1 activation and osteoblast differentiation. Therefore, it was concluded that mTORC1 signaling is involved in bone regeneration mediated by ZA [74]. Another signaling pathway that plays a role in osteogenesis of BPs is BMP cascade. It has been demonstrated that controlled-release of alendronate (ALN) potentiates rat BMMSCs towards osteogenesis by increasing pSmad1/5. Additionally, ALN can decrease p38, NF-kB and ERK in RANK signaling (Fig. 4) which contributes to inhibition of osteoclastogenesis [75]. Activation of other pro-osteogenic signaling pathways improves osteogenic activity of BPs. One small molecule with adjunctive osteogenic properties with BPs is SM. Simultaneous application of SM and ALN has demonstrated an improvement in osteogenic differentiation compared to single treatment of each agent in rat adipose-derived MSCs (AD-MSCs) [76]. Combination of recombinant human BMP-2 and ALN is another method to ameliorate osteogenesis. Serial treatment with these two agents increases osteogenic transcription factors in MC3T3-E1 mouse pre-osteoblast cells. Optimal results are obtained when BMP treatment is followed by ALN [77]. Physical traits of the extracellular matrix are known to influence the outcome in tissue regeneration. Jiang et al. investigated the relationship between matrix stiffness and ALN on osteogenic differentiation. They synthesized ALN loaded hydrogels with a variety rate of stiffness. Rat BMMSCs were used to evaluate in vitro osteogenic differentiation. After 7 days of culture, a positive relationship between ALN concentration and bone tissue regeneration level was observed. In addition, at the same ALN concentration, osteogenesis was more enhanced in the hydrogels with higher rates of stiffness. There seems to be a synergistically positive correlation between ALN and substrate stiffness regarding osteogenic differentiation. Therefore, it is considered a potential method for boosting osteogenic differentiation of BPs [78].

### Dexamethasone

Dexamethasone (DEX) is a synthetic corticosteroid agent which is known as an inducer of osteogenic differentiation. DEX-mediated bone formation is thought to be linked with Wnt and MAPK signaling pathways in stem cells derived from human exfoliated deciduous teeth (SHEDs) [79]. Treatment with DEX results in upregulation of a LIM-domain protein known as FHL-2. This is apparently due to binding of DEX to a glucocorticoid response element in the promoter region of FHL-2. Moreover, when Wnt3a (a member of the canonical Wnt signaling pathway) is present, FHL-2 binds to β-catenin (Fig. 2). This binding eases transportation of β-catenin into the nucleus, thus contributing to downstream osteogenic gene expression. Moreover, DEX administration has been found to increase expression of MAPK phosphatase. This causes ERK 1/2 phosphorylation followed by dephosphorylation of Runx2 transcription factor and therefore leads to osteogenic differentiation [80].

DEX has been reported to enhance response of stem cells to BMP-2 mediated osteogenic differentiation. Recently, BMP-2 and DEX have demonstrated augmented osteogenic differentiation in muscle-derived stromal cells. Animal experiment has shown similar results. In vivo implantation of BMP-2 and DEX showed synergistically enhanced ectopic bone formation compared to implants containing BMP-2 only [81]. Another study focusing on BMP-2/DEX combination has reported that combination treatment increases canine MSCs mineralization. However, these results were not seen in canine iPSC-derived MSCs [82]. Although BMP-2 seems to be an ideal agent for adjuvant treatment with DEX, its delivery to the site of treatment is related with various side effects such as tumorigenesis and immunological reaction. To overcome this, BMP synthetic peptides are used. BMP-2 peptides are peptides shaped based on the BMP-2 “knuckle.” The BMP-2 knuckle epitope is the part of BMP-2 through which it binds to type 2 BMP receptors. BMP-2 peptides can cause regeneration of bone both in vitro and in vivo when used with biomaterials. Zhou et al. utilized a co-delivery system of BMP-2 peptide and/or DEX to induce osteogenic differentiation of BMMSCs. The results indicated that DEX and BMP peptide can synergistically enhance osteogenic differentiation of stem cells. In vivo ectopic bone formation was also upregulated in animals treated with both BMP-2 peptide and DEX [83, 84]. Hydroxyapatite nanoparticles (nHA) are another option to be used as adjuvant treatment of DEX during osteogenesis. nHA and DEX-containing scaffolds were utilized to enhance osteogenesis in AD-MSCs. Induction of osteogenic differentiation by these scaffolds revealed a synergistically promoted differentiation in combination therapy of stem cells rather than single treatment. Therefore, combination of nHA and DEX can be a possible means of enhancement of DEX-mediated osteogenesis [85].

### Melatonin

Melatonin (MLT) is a hormone secreted from the hypothalamus which is involved in regulation of the circadian rhythms [86]. Application of MLT has recently been found to be related with enhancement of osteogenesis and abolishment of osteoclast formation [87]. Moreover, MLT is able to induce osteogenesis coupled with angiogenesis in rat BMMSCs when cultured in osteogenic medium [88]. MLT can restore osteogenic differentiation of human BMMSCs undergone oxidative stress by hydrogen peroxide through activation of AMPK signaling pathway. This was marked by an increase in FOXO3a and RunX2 gene expression after addition of MLT (Fig. 3). Incubation of cells with an AMPK inhibitor abolished these effects. Therefore, AMPK pathway seems to be involved in the osteogenic differentiation of MLT [89]. Another study indicates that MLT can differentiate stem cells into osteoblasts via the G-protein coupled receptor MT2 and MAPK signaling pathway. However, this was abrogated when PKA and PKC specific inhibitors were used. Thus, PKA and PKC seem to be involved in osteoblast differentiation of MLT [86]. Another pathway found to play a role in MLT-induced bone regeneration is Neuropeptide signaling. After treatment of rat BMMSCs with MLT, osteogenesis was abolished when a NPY signaling inhibitor was administered. It was found that NPY and NPY1 receptor were involved in the process of osteogenesis (Fig. 3). It was further found that treatment with MLT can result in bone regeneration in rats with femoral fractures. Similar to the in vitro study, administration of an NPY1 receptor inhibitor abolishes osteogenesis in animal models [46]. In the recent years, MLT has been investigated as an agent for treatment of osteoporosis. Zhang et al. has demonstrated that MLT can promote osteoblastogenesis through reduction in Nucleotide-binding domain and the leucine-rich repeat pyrin 3 domain (NLRP3) inflammasome activity in ovariectomized rats. NLRP3 is a proinflammatory agent which suppresses bone formation. This agent is inhibited by activated β-catenin. In this study, they observed that when the Wnt signaling antagonist DKK-1 was employed, osteogenesis was perished. Further investigations showed that application of MLT resulted in lower GSK3β expression and higher active β-catenin. Thus, canonical Wnt signaling was considered the mechanism by which MLT enhanced osteogenesis [87]. Another mechanism through which MLT can act against osteoporosis is through reversing TNFα effects. TNFα can disrupt osteogenesis and therefore contribute to inflammation-related osteoporosis. TNFα-mediated disruption is due to interfering with BMP-Smad signaling. It was shown that TNFα addition resulted in lower Smad1 protein and pSmad1 levels. However, administration of MLT was resulted in restored BMP-Smad activity and pSmad levels. Further investigations revealed that MLT attenuated TNFα mediated Smad1 ubiquitination and degradation. Smad1 ubiquitination is mainly regulated by Smad ubiquitination regulatory factor 1 (Smurf1), which is overexpressed by TNFα. Utilization of MLT inhibits TNFα-induced Smurf1 overexpression and thus stabilized Smad1. [90]. MLT can also affect MAPK signaling pathways during osteogenesis. Activating p38 and ERK1/2 MAPK is suggested as a necessary mechanism for MLT-induced osteogenic differentiation of rabbit DPSCs. Furthermore, MLT inhibits NF-kB signaling, abolishing osteoclast differentiation by hindering RANK signaling (Fig. 4) [91]. It seems that circular RNAs also affect MLT-related osteogenic differentiation. MLT can shift human BMMSCs towards osteogenic lineage by altering gene expression of several circular RNAs. Subsequently, one of these circular RNA can induce osteogenic gene expression through interactions with miR-3653-3p [92].

Although MLT is considered as a potential osteogenic agent, its water insolubility has restrained its intracellular uptake. Therefore, improving MLT water solubility may contribute to a wider application in the clinic. Cyclodextrin is an oligosaccharide which has previously shown improved water solubility of simvastatin. Terauchi et al. used 2-hydroxypropyl beta-Cyclodextrin to form an inclusion complex with MLT. 2-hydroxypropyl beta-Cyclodextrin increased intracellular concentration of MLT and its metabolites and enhanced osteogenesis in MC3T3-E1 cells. Therefore, utilization of 2-hydroxypropyl beta-Cyclodextrin with MLT may be a promising method for bone regeneration [93].

Application of other molecules alongside MLT has also been shown to contribute to enhancement of osteogenesis. Maioli et al. used a combination of hyaluronic, butyric and retinoic acid with MLT to induce differentiation. The combination was found to upregulate expression of osteogenic specific genes in a synergistic manner. This synergistic effect is possibly through upregulation of vascular endothelial growth factor. In addition, MLT + Retinoic acid (RA) + Butyric acid (BU) + Hyaluronic acid (HA) medium exhibited higher levels of osteogenic gene expression compared to a traditional dexamethasone-based osteogenic medium. The results from this study suggest that adding the combination of RA, BU and HA to MLT can increase MLT induced osteogenic differentiation [94]. Addition of BMP-4 to MLT is another way through which MLT osteogenesis can be improved. Mouse C2C12 cells seeded with BMP-4 and MLT showed higher rate of osteogenic differentiation than that of BMP-4 alone [86].

### Metformin

Compelling evidence suggests that metformin, a biguanide widely known as a type 2 diabetic agent, can effectively lower risk of bone fractures. Several studies have demonstrated that application of metformin can contribute to proliferation and osteogenic differentiation of stem cells [95]. Gu et al. studied the effects of metformin on osteogenic differentiation of human chorionic villous MSCs (CV-MSCs). Administration of metformin was consistent with upregulation of several osteogenic specific genes after 7 and 14 days as well as tissue mineralization. These results suggest that application of metformin successfully leads CV-MSCs into osteogenic lineages. In order to assess its effects on adipogenic differentiation, metformin was added into an adipogenic medium of cultured CV-MSCs. It was found that expression levels of proliferators-activated receptor γ (PPARγ) were notably downregulated in metformin group. This suggests that metformin administration reduces adipocyte formation of CV-MSCs and increases osteogenic differentiation [96]. It seems that metformin-induced osteogenic differentiation is mainly via activation of AMPK [97]. AMP-activated protein kinase is a serine-threonine kinase consisting of 3 subunits responsible for switching on cellular catabolic pathways. AMPK needs to be phosphorylated at one of its subunits by the main AMPK kinase known as LKB1 to be activated. AMPK activation has recently been found to associate with osteogenic differentiation [97–99]. Utilization of plasmids containing kinase dead LKB1 gene results in reduced AMPK phosphorylation and osteogenic differentiation in hiPSC-derived MSCs undergoing differentiation in metformin-containing medium. On the other hand, in wild type induced-pluripotent-derived mesenchymal stem cells, opposite results were achieved [99]. Furthermore, using an AMPK inhibitor inhibits metformin-induced osteogenic differentiation [97]. Activation of AMPK by metformin has been found to relate to inhibition of GSK3β, which is a crucial member of Wnt signaling pathway. Metformin inhibited GSK3β activity results in β-catenin protein accumulation and engagement of canonical Wnt signaling. Furthermore, utilization of an AMPK inhibitor reduces GSK3β phosphorylation in the osteogenic differentiation of hBMMSCs [100]. Moreover, metformin-induced AMPK activation can directly influence RunX2 expression. It has recently been found that AMPK can directly phosphorylate RunX2 and metformin mediated activation of AMPK can reduce RunX2 phosphorylation loss [45]. Although AMPK activation seems to be the main mechanism of metformin induced osteogenic differentiation, Nitric Oxide and BMP-2 expression are also considered possible pathways. However, the exact effect of metformin on BMP-2 expression remains controversial [96]. Also, metformin exerts osteogenic effects through p38 and ERK1/2 signaling pathways. It has been shown that high glucose damages differentiation capacities of stem cells. Culturing of periodontal ligament stem cells which have been under high glucose condition with metformin restores their osteogenic potential through downregulation of p38 and ERK1/2 phosphorylation [101].

Since metformin is a hydrophilic agent, its cellular uptake depends on cell membrane organic cation transformers (OCTs). Jofi et al. hypothesized that presence of functional OCTs would be essential in order to induce metformin-mediated osteogenesis of cells. To this end, they harvested MSCs from human umbilical cord Wharton’s jelly (UC-MSCs) and studied whether or not OCTs are found in the membrane of these cells and whether they can affect osteogenic differentiation induced by metformin. It was observed that different OCT isoforms were present in UC-MSCs. After subjecting these cells to different doses of metformin, the osteogenic involved LKB1/AMPK signaling pathway was activated. Furthermore, cell lines treated with metformin revealed an increased osteogenic commitment with upregulation of bone specific genes and mineralized nodules. However, these results were abolished when the pan-OCT inhibitor quinidine was used. This suggests that functional presence of OCT is required for achieving effective osteogenesis by metformin [95].

### Phenamil

Phenamil is a derivative of the diuretic agent, amiloride, which targets sodium channels in the cell membrane and the Na/H exchanger for their function. Other than its diuretic effects, Phenamil is known to induce osteogenic differentiation through BMP/Smad signaling pathway [102, 103]. Using phenamil as an osteogenic agent has been investigated both in long-term and short-term assays. Lo et al. investigated the effects of short-term (12 h) administration of the small molecule phenamil on osteogenesis of MC3T3-E1 osteoblast-like cells. It was found that proliferation of cells treated with long-term phenamil was decreased compared to short-term after 3 days of culture. It was found that no significant difference was observed in osteogenic markers in short-term and long-term treatment groups. However, upregulation of osteogenic markers was observed in both groups compared with control [104]. Other studies have revealed that phenamil can enhance osteogenesis in AD-MSCs and human DPSCs as well [14, 105].

Phenamil is known to induce osteogenic differentiation through BMP/Smad signaling pathway. Phenamil-induced osteoblastogenesis is related with upregulated Trib3 expression. Tribble’s homologs family members (Tribs) are involved in different cellular actions including cell differentiation. Although the mechanism by which phenamil exerts Trib3 gene expression during osteogenesis is not fully understood, which is thought to be through ion channel function inhibition. In another study regarding vascular remodeling, phenamil induced Trib3 gene expression by acid-sensing ion channel inhibition. Increase in Trib3 expression downregulates the BMP antagonist, Smurf1, which is consistent with enhanced Smad protein levels and osteogenic genes expression. In addition, phenamil-mediated expression of Trib3 abrogates BMP-2-induced adipogenesis by suppression of PPARγ expression. Moreover, phenamil upregulates BMP-2 expression in hBMMSCs. Therefore, phenamil induces osteogenesis by engagement of BMP-Smad pathway and therefore attenuates adipogenesis [106].

Since phenamil has been proven to increase BMP level in stem cells, Fan et al. investigated whether incubation of cells with Phenamil and BMP-2 can enhance osteogenic differentiation. It was found concomitant treatment of AD-MSCs with Phenamil and BMP-2 can accelerate osteogenic differentiation [13]. Addition of Phenamil to BMP-2 showed better quality of bone formation. Phenamil inhibited BMP-2 induced cyst-like bone formation, inflammatory soft tissue swelling and adipogenesis [106]. Mouse calvarial defect models were used to assess bone regeneration of adjoined treatment of BMP-2 and phenamil. AD-MSCs were utilized for the experiment. It was reported that phenamil + BMP-2 group showed a higher level of osteogenic marker expression and bone formation [13]. These findings suggest that BMP-2 can be an effective adjuvant treatment during osteogenic differentiation of Phenamil. Since phenamil induces osteogenic differentiation through BMP/Smad signaling, downregulation of BMP antagonist (Noggin) might present advantageous osteogenic effects. It was demonstrated that application of phenamil with simultaneous shRNA induced suppression of Noggin (Nsh-RNA) positively regulates osteogenic differentiation. This finding was further confirmed in healing of mouse calvarial defects. Co-treatment of phenamil and Nsh-RNA leads to enhanced bone regeneration [14]. Strontium ion (Sr^2+^) is another agent which has been used together with phenamil to induce osteogenesis. Co-delivery of Sr^2+^ and phenamil resulted in enhanced osteo/odontogenesis of stem cells. It was further found that co-incubation with Sr and phenamil resulted in higher Smad1/5/8 phosphorylation which possibly contributed to this additive effect. It has been shown that co-treatment of animals with mal-calcification conditions resulted in higher level of new hard tissue. These results suggest that co-delivery of phenamil and Sr^2+^ may be a possible strategy for adjuvant treatment for bone tissue engineering [105].

### Parathyroid hormone

Parathyroid hormone (PTH) is a bone anabolic agent which has been approved for treatment of osteoporosis. Application of PTH results in increased bone strength and mass. Moreover, utilization of PTH is known to enhance bone regeneration through increased osteocyte activity and osteogenic differentiation of MSCs [107]. Although PTH is a common agent used to prevent osteoporosis, long-term administration of high dose PTH has been related with disruption of osteoblast function. Also, animal studies have shown that this agent can increase risk of osteosarcoma. These results are not observed with PTH-related Peptide (PTH-rP). This makes PTH-rP a more suitable candidate for osteoporosis prevention compared with PTH. PTH-rP and PTH both act through the same PTH receptor (PTH1R) and have the same effects in regards to phosphate and calcium regulation [108]. Zhang et al. studied the effects of PTH-rP on osteogenesis of MSCs. Their results showed that addition of PTH-rP to the osteogenic medium contributed to enhancement of osteogenic differentiation and inhibition of adipogenic differentiation [108].

Signaling of PTH occurs through a G αs/αq-protein-coupled receptor and sequentially cAMP and PKA signaling. Although most studies have shown cAMP as an osteoblastogenic factor, some have shown contradicting results. These results are likely due to differences in the mechanisms through which cAMP is modulated, concentration level of cAMP and the duration of elevated cAMP level [29]. It seems that PTH can induce osteogenic differentiation of stem cells via activation of BMP signaling pathway as well. It has been shown that one injection of PTH in mouse model increases phosphorylated Smad1. Application of PTH inhibits the effect of Noggin on pSmad1 levels. Upon binding of PTH to type 1 PTH receptors (PTHR1), which are a type of G-protein coupled receptors, a complex consisting of PTHR1 and Low-density lipoprotein receptor-related protein 6 (LRP 6) is formed and endocytosed. LRP 6, which is considered a member of the Wnt signaling pathway, has several mutual antagonists with BMP receptors. It has been found that LRP 6 deletion on its own increases pSmad levels. Therefore, it is believed that the crosstalk between PTHR1 and BMP signaling occurs mainly due to endocytosis of PTHR1/LRP6 complex and easing access of BMP ligands to BMP receptors. Altogether, PTH-induced osteogenic differentiation seems to be mediated through engagement of BMP-Smad signaling pathway and endocytosis of PTHR1/LRP6 seems to be the likely mechanism for this effect [109]. Stimulation of PTH receptors is also known to activate PKA and PKC pathways. A recent study investigated whether administration of intermittent PTH can induce osteogenic differentiation via PKA and/or PKC pathways. It was reported that intermittent treatment further increased PKC activity compared to continuous treatment. To investigate whether activation of PKC was related with osteogenic differentiation, a PKC inhibitor was employed. Administration of the PKC inhibitor resulted in lower osteogenic specific gene expression. This suggests that osteogenic differentiation by intermittent application of PTH is related with PKC activation [43]. To investigate possible additive treatments, Weng et al. recently conducted a study on the combination of PTH and vitamin K (Vit K). It was observed that combination therapy not only prevents bone loss, but also promotes bone formation in osteoporotic tissue. Furthermore, differentiation assay revealed that combination of PTH and Vit K enhances BMMSCs osteogenic differentiation compared with either of the substances alone and upregulates in vivo bone mineralization [107].

### Resveratrol

Resveratrol is a natural polyphenol which is found in various plants and red wine and has bone-protective characteristics [110, 111]. Resveratrol is a known inducer of bone formation both in vitro and in vivo [112]. Osteogenic differentiation of resveratrol is enhanced when used alongside alendronate or zoledronic acid. Therefore, it is considered as a candidate for adjunctive treatment with bisphosphonates during osteogenesis [113]. Resveratrol is considered as a possible treatment for bone metabolism impairment due to inflammation. Ma et al. investigated bone protective effects of resveratrol in LPS-induced degradation of MC3E3-T1 osteoblast cells. In order to induce bone degradation, the cells were first cultured in medium containing different cultures of LPS for 24 h. They showed that post-treatment with resveratrol increased cellular viability. Furthermore, LPS-induced inhibition of osteogenic differentiation was reduced with resveratrol treatment. Resveratrol could reverse LPS-mediated inhibition of Silent information regulator 1 (Sirt1) and PGC-1alpha expression. Since these molecules are necessary for mitochondrial function, restoration of expression of these genes might be the mechanism through which resveratrol treatment enhanced LPS-mediated osteogenic inhibition [111].

Previously, it was known that resveratrol can induce osteogenic differentiation possibly through estrogen receptor-mediated activation of ERK1/2 MAPK signaling pathway. Also, resveratrol can abolish osteoclast differentiation by inhibition of RANKL signaling in DPSCs [110, 114]. However, other signaling pathways have been found to play roles in resveratrol-mediated bone differentiation. Tseng et al. demonstrated that utilization of sirtinol, as Sirt1 inhibitor, partly inhibited bone regeneration mediated by resveratrol in human ESC-derived MSC progenitors. Sirt1 is a nicotinamide adenine dinucleotide dependent deacetylase which activates various transcription factors such as class O subfamily of forkhead (FOXO), NF-kB and p53. It was found that unlike the previous assumption that resveratrol exerts osteogenic effects mainly through estrogen signaling, the main mechanism of resveratrol-mediated osteogenesis lies in Sirt1/FOXO3A pathway. FOXO3A is considered a member of FOXO family which regulates stress resistance genes transcription [115] (Fig. 4). Sirt1 can also repress PPARγ through crosstalks with nuclear receptor co-receptor (NCor) which is a PPARγ co-factor in canine AD-MSCs. This suggests that resveratrol can deviate cellular differentiation path from adipogenesis to osteogenesis [116]. Recently, Wang et al. found that activation of Sirt1 by resveratrol results in upregulated Sirt1- B lymphoma Mo-MLV insertion region 1 homolog (Bmi1) binding in mouse BMMSCs. Bmi-1 and Sirt1 are polycomb group proteins. This means they share the same target genes. Bmi-1 regulates various stem cell characteristics such as proliferation, senescence and cell cycle. Consequently, knockdown of Bmi1 suppressed bone volume in mice with Sirt1 overexpression. Thus, resveratrol mediates its osteogenic effects at least partly through Sirt1-Bmi1 [117]. Wnt signaling pathway has also been reported to be accountable for osteogenic differentiation by resveratrol and incubation with resveratrol engaged Wnt/Gsk-3b/β-catenin signaling pathway during osteogenesis [118].

### Vitamin D3

Supplementation of Vitamin D3 (Vit D3) is recommended in all treatment and prevention strategies of osteoporosis. A wide range of bone catabolic and anabolic mechanisms are known to be mediated through the active form of vit D3 which is 1,25-dihydroxyvitamin D3 [119]. It acts as an important factor for maintaining calcium and phosphate levels as well as an inducer of osteogenic differentiation. There are two vit D3 receptors present in human osteoblast cells: the canonical nuclear vitamin D receptor (VDR) and a protein disulfide isomerase family A member 3 (Pdia3) on the plasma membrane. Both receptors are known to have key roles in development of bone tissue. Chen et al. studied the interaction between VDR and BMP signaling and found that combination of BMP-2 and Vit D3 has a synergistic effect on expression of osteogenesis of MC3T3-E1 cells. Si-RNA mediated suppression of Pdia3 and VDR abrogated the mentioned enhancement of osteogenesis and it was found that VDR has a more prominent role in regards to this synergistic effect [120]. Adrenocorticotropic hormone (ACTH) is another agent with potential additive effects on Vit D3 mediated bone formation. Combination of ACTH and 1,25-dihydroxyvitamin D3 synergistically enhanced osteogenesis of hBMMSCs [121]. Vitamin MK-7, a type of Vitamin K2, has also been considered a possible co-treatment agent. Gigante et al. investigated the effects of Vitamin MK-7 on Vit D3 induced osteogenic differentiation of hBMMSCs. It was found that combination therapy resulted in higher levels of osteogenic specific genes and proteins. Therefore, Vitamin MK-7 is a suitable agent for adjuvant treatment with Vit D3 [122].

Oxidative stress is one of the notable obstacles in the process of osteogenesis. Xiong et al. demonstrated that utilization of Vit D3 can promote osteogenesis of mouse BMMSCs under oxidative stress induced by high glucose in diabetic patients. They found that Vit D3 can inactivate FOXO1 through PI3K/Akt signaling pathway and binding of FOXO1 with β-catenin is crucial for FOXO1-mediated transcription. This suggests that Vit D3 can enhance osteogenesis in oxidative stress of high-glucose condition through Wnt-β-catenin signaling pathway [123]. Zhou et al. investigated the effects of Vit D3 on osteogenic differentiation of human AD-MSCs in H_2_O_2_-induced oxidative stress. While H_2_O_2_ administration reduced calcium deposition and mRNA level of osteogenic genes, Vit D3 restored this injury. Application of Vit D3 was consistent with upregulation of Sirt1/FOXO1 axis. When the Sirt1 inhibitor, sirtinol, was utilized, Vit D3-induced enhancement of osteogenesis in oxidative condition was attenuated. Therefore, Sirt1/FOXO1 enhancement appears to be responsible for improvement of osteogenic differentiation by Vit D3 under oxidative stress [124].

### T63

T63 is a small molecular compound that has recently been identified as a promoter of osteogenesis and suppressor of adipogenesis in MC3T3-E1 cells. T63-mediated osteogenic differentiation seems to take place through activation of two intracellular signaling pathways: 1- Canonical Wnt signaling is activated when T63 is administered. Incubation of cells with T63 results in upregulation of β-catenin and phosphorylation of GSK-3β. In addition, when cells were treated with the Wnt inhibitor, DKK-1, osteogenic differentiation was attenuated. 2- Utilization of T63 resulted in increased level of pSmad1/5/8 and BMPs 2, 4 and 7. Thus, T63 regulates osteogenesis by activating BMP signaling. Furthermore, application of BMP-specific antagonist Noggin abrogated osteogenesis of T63. This suggests that BMP signaling possibly acts as the upstream pathway in activation of Wnt signaling by T63 [125].

### THQ-1a and PP-9

Tetrahydroquinolines (THQs) and pyrazolopyrimidines (PPs) are two classes of molecules which have previously been found to enhance Wnt signaling pathway [126].

Chen et al. investigated possible roles of THQs and PPs in promoting osteogenic differentiation. They synthesized 24 derivatives (12 THQs and 12 PPs) and studied their osteogenic potentials on C2C12 myoblast cells. After culturing cells with these agents, it was found that one of the THQ derivatives, named THQ-1a and one of the PPs, named PP-9 were the most potent osteogenic inducers. Incubation of C2C12 myoblast cells with both of the above-mentioned substances was consistent with increase in β-catenin protein levels after 3 days. Thus, application of PP-9 and THQ-1a induced osteogenic differentiation possibly through activation of Wnt signaling [127].

### Smoothened agonists

Smoothened is a transmembrane signaling transducer of Hedgehog signaling pathway and its deletion is related with diminished bone formation in mice. Kashiwagi et al. studied the effects of a Smoothened agonist (566,660, Calbiochem, Darmstadt, Germany) (SAG) on bone formation in mice. They found that local single-shot SAG injection increased callus formation during bone regeneration. To examine the effect of Hh agonism in fracture healing, a single dose of SAG was delivered to the site of defect in mice with tibial fractures. It was observed that SAG application caused an increase in mineralization of calluses. Although the cellular morphology was the same between treatment and control group, an increase in the number of cartilaginous cells was found in the former. Immunostaining of the cells showed an increase in proliferation as the mechanism of soft callus growth. Furthermore, exogenous Hh agonism resulted in an advance in formation of hard callus [128]. Moreover, the effects of SAG on osteoblast differentiation were also tested in another study by kanke et al. It was found that after mesoderm induction, culturing mouse ESCs, mouse iPSCs and hiPSCs with SAG successfully upregulated osteogenic specific gene and tissue mineralization [129].

Purmorphamine is another agonist of the smoothened receptor and thus activator of Hh signaling pathway [130]. Activation of this pathway by purmorphamine induces osteogenic differentiation [15]. Wöltje et al. studied the effects of rat BMMSCs culturing with purmorphamine. It was found that purmorphamine can lead MSCs towards osteoblast lineages and upregulate tissue mineralization [131]. In another study, the effects of purmorphamine on DPSCs were evaluated. Incubation of cells with purmorphamine resulted in upregulation of both early and late-stage osteogenic specific genes and proteins and effectively shifted cells towards osteoblast lineage [15].

### Rapamycin

Recently, the mTOR inhibitor rapamycin has been found to induce osteogenic differentiation. Through affecting the PI3K/AKT/mTOR signaling pathway, rapamycin can alter RunX2 and BMP-2 levels and thus contribute to osteogenic differentiation. Carpentieri et al. investigated rapamycin’s potential in inducing osteoblast differentiation of blood-derived stem cells (BDSCs). After ten days, it was found that BDSCs’ morphology had altered into osteoblast like cells. Furthermore, there were Calcium-containing granules and Calcium phosphate containing vesicles in the mitochondria which was a sign of bone mineralization [132].

## Conclusion and future perspectives

Small molecules, as key players in tissue engineering, are a major factor in the field of osteogenic differentiation and an important component in improving osteogenesis. Activation of intracellular osteogenic signaling cascades is the method through which these agents induce osteoblast differentiation. For majority of these agents, more than one signaling pathway plays a role in osteogenic differentiation. For example, BMP/Smad and canonical Wnt signaling are both crucial in simvastatin-mediated osteogenesis. Additionally, both BMP/Smad signaling and OPG/Rankl signaling influence differentiation outcomes induced by bisphosphonates. However, some pathways such as ERK ½ and p38 have shown contradictory results during different molecules-mediated osteoblast differentiation. For instance, while ERK ½ positively correlates with DEX-induced osteogenesis, this pathway has been found to have a negative correlation with statin-mediated osteogenesis. These results point out the need for more extensive research on the mechanisms by which small molecules induce osteogenic differentiation. Of the currently studied small molecules, Dex, Vit-D3, and Simvastatin have been evaluated more extensively and yield the greatest potential for application in the clinic. Moreover, it seems that combination of small molecules together or with growth factors is needed to achieve a more specific osteogenic differentiation of stem cells. Several cell lines including BMMSCs, AD-MSCs, DPSCs, and Amniotic epithelial cells have been previously differentiated to the osteogenic cells by using small molecules [133]. Employing the right stem cell line and small molecule seems to be the key players in achieving optimal results in order for effective translation into the clinic. In order to achieve this goal, the mechanisms for small molecule-mediated osteogenesis need to be further studied.

Another necessary factor for successful tissue regeneration is scaffolds that mimic the natural cellular niche and regulate cell proliferation and differentiation. The incorporation of several small molecules in natural and synthesized scaffolds such as Polyethylene glycol diacrylate (PEGDA), Poly (lactic-co-glycolic acid) (PLGA), calcium phosphate, and gelatin has been previously studied [31, 134–136]. However, designing the optimal scaffolds that release small molecules for bone regeneration is still considered a challenge. Also, further studies need to be done in regard to the interaction of small molecules with scaffolds [17].

Although natural growth factors such as BMP have been previously studied in fracture healing studies, small molecules have rarely been utilized to do so [137]. The results from a clinical trial by shim et al. indicate that the combination of teriparatide, which is a recombinant human PTH, and Wharton’s jelly stem cells result in better vertebral bone fracture healing by enhancing the bone architecture compared to stem cells alone [138]. Pretreatment of stem cells with small molecules before injection into the site of fracture is one of the methods which needs to be evaluated in the future studies. In order to have a clinical insight into the basic research, there needs to be an interdisciplinary effort of experts in medicine, pharmacology, materials sciences, and nanotechnology. An in-depth understanding of the interaction between small molecules and scaffolds as well as the signaling pathways governing small molecule-mediated bone regeneration can lead to effective, inexpensive, and accessible bone tissue engineering and potentially pave the way for the treatment of bone defects and nonunion fractures.

## Data Availability

Not applicable.

## Abbreviations

BMP: Bone morphogenic protein
FZD: Frizzled
GSK-3: Glycogen synthase kinase 3
Dvl: Dishevelled
PKC: Protein kinase C
LRP5/6: Lipoprotein receptor-related proteins 5 or 6
TGF-β: Transforming growth factor-beta
FHL-2: Four and a half LIM domains protein 2
MSCs: Mesenchymal stem cells
PTCH: Patch
Smo: Smoothened
OPG: Osteoprotegerin
TRAF: Tumor necrosis factor receptor-associated factor
ROCK: Rho-associated coiled coil-containing protein kinase
mDia: Mammalian diaphanous
LIMK: LIM motif-containing protein kinase
GLI: Glioma-associated oncogene transcription factor
MAPKs: Mitogen-activated protein kinases
ERK 1/2: Extracellular receptor kinase 1/2
PKA: Protein kinase A
AMPK: Adenosine monophosphate-activated kinase
NPY: Neuropeptide Y
RANKL: Receptor activator of NF-kB ligand
mTOR: Mechanistic target of rapamycin
ER-alpha: Estrogen receptor-alpha
GPER-1: G protein coupled estrogen receptor-1
SM: Simvastatin
ESCs: Embryonic stem cells
BMMSCs: Bone marrow mesenchymal stem cells
PBMCs: Peripheral blood mononuclear cells
DKK-1: Dickkopf-1
VEGF: Vascular endothelial growth factor
FGF-2: Fibroblast growth factor
DPSCs: Dental pulp stem cells
PTH: Parathyroid hormone
Nsi-RNA: Si-RNA targeting Noggin gene
PSCs: Pluripotent stem cells
BPs: Bisphosphonates
ZA: Zoledronic acid
ALN: Alendronate
AD-MSCs: Adipose-derived MSCs
DEX: Dexamethasone
SHEDs: Stem cells derived from human exfoliated deciduous teeth
nHA: Hydroxyapatite nanoparticles
MLT: Melatonin
NLRP3: Nucleotide-binding domain and the leucine-rich repeat pyrin 3 domain
RA: Retinoic acid
BU: Butyric acid
HA: Hyaluronic acid
CV-MSCs: Chorionic villous MSCs
PPARγ: Proliferators-activated receptor γ
OCTs: Organic cation transformers
UC-MSCs: MSCs from human umbilical cord Wharton’s jelly
Tribs: Tribble’s homologs family members
Sr^2+^: Strontium ion
PTH-rP: PTH-related peptide
PTHR1: Type 1 PTH receptors
Vit K: Vitamin K
FOXO: Class O subfamily of forkhead
NCor: Nuclear receptor co-receptor
Bmi1: B lymphoma Mo-MLV insertion region 1 homolog
Vit D3: Vitamin D3
VDR: Vitamin D receptor
Pdia3: Protein disulfide isomerase family A member 3
ACTH: Adrenocorticotropic hormone
THQs: Tetrahydroquinolines
PPs: Pyrazolopyrimidines
SAG: Smoothened agonist
BDSCs: Blood-derived stem cells
